# Automatic acoustic recognition of pollinating bee species can be highly improved by Deep Learning models accompanied by pre-training and strong data augmentation

**DOI:** 10.3389/fpls.2023.1081050

**Published:** 2023-04-14

**Authors:** Alef Iury Siqueira Ferreira, Nádia Felix Felipe da Silva, Fernanda Neiva Mesquita, Thierson Couto Rosa, Victor Hugo Monzón, José Neiva Mesquita-Neto

**Affiliations:** ^1^ Instituto de Informatica, Universidade Federal de Goias, Goiania, Goias, Brazil; ^2^ Laboratorio Ecologıa de Abejas, Departamento de Biologıa y Quımica, Facultad de Ciencias Basicas, Universidad Catolica del Maule, Talca, Chile

**Keywords:** buzz-pollinated crops, ecosystem services, crop pollination, machine-learning, blueberry crops

## Abstract

**Introduction:**

Bees capable of performing floral sonication (or buzz-pollination) are among the most effective pollinators of blueberries. However, the quality of pollination provided varies greatly among species visiting the flowers. Consequently, the correct identification of flower visitors becomes indispensable to distinguishing the most efficient pollinators of blueberry. However, taxonomic identification normally depends on microscopic characteristics and the active participation of experts in the decision-making process. Moreover, the many species of bees (20,507 worldwide) and other insects are a challenge for a decreasing number of insect taxonomists. To overcome the limitations of traditional taxonomy, automatic classification systems of insects based on Machine-Learning (ML) have been raised for detecting and distinguishing a wide variety of bioacoustic signals, including bee buzzing sounds. Despite that, classical ML algorithms fed by spectrogram-type data only reached marginal performance for bee ID recognition. On the other hand, emerging systems from Deep Learning (DL), especially Convolutional Neural Networks (CNNs), have provided a substantial boost to classification performance in other audio domains, but have yet to be tested for acoustic bee species recognition tasks. Therefore, we aimed to automatically identify blueberry pollinating bee species based on characteristics of their buzzing sounds using DL algorithms.

**Methods:**

We designed CNN models combined with Log Mel-Spectrogram representations and strong data augmentation and compared their performance at recognizing blueberry pollinating bee species with the current state-of-the-art models for automatic recognition of bee species.

**Results and Discussion:**

We found that CNN models performed better at assigning bee buzzing sounds to their respective taxa than expected by chance. However, CNN models were highly dependent on acoustic data pre-training and data augmentation to outperform classical ML classifiers in recognizing bee buzzing sounds. Under these conditions, the CNN models could lead to automating the taxonomic recognition of flower-visiting bees of blueberry crops. However, there is still room to improve the performance of CNN models by focusing on recording samples for poorly represented bee species. Automatic acoustic recognition associated with the degree of efficiency of a bee species to pollinate a particular crop would result in a comprehensive and powerful tool for recognizing those that best pollinate and increase fruit yields.

## 1 Introduction

Highbush blueberry (*Vaccinium corymbosum* L.: Ericaceae) requires insect-mediated pollination to enhance fruit quality (Brewer and Dobson, 1969; Benjamin and Winfree, 2014). The flow of pollen among flowers promoted by biotic vectors increases fruit set and berry size (Dogterom et al., 2000; Nicholson and Ricketts, 2019). However, the specialized morphology of blueberry flowers, characterized by the presence of poricidal anthers and narrow/bell-shaped corollas, limits pollen access to certain floral visitors (Buchmann, 1983; De Luca et al., 2013; Corbet and Huang, 2014; Russell et al., 2017; Cooley and Vallejo-Marín, 2021). To extract pollen efficiently, a floral visitor needs to vibrate a blueberry flower such that the vibrations are transmitted to the pollen within the anthers, stimulating it to leave *via* small openings. The vibrations produce a particular audible sound that gives the name to this phenomenon: buzz-pollination or sonication (Buchmann, 1983). Probably because of this, bees that perform floral sonication are among the most effective pollinators of blueberries (Cane et al., 1985; Javorek et al., 2002; Nicholson and Ricketts, 2019). In fact, only a subset of all visitors can actually pollinate (Schemske and Horvitz, 1984; Kandori, 2002). The quality of the pollination provided varies greatly and is partially related to the taxonomic identity of the flower visitors visitors (Nunes-Silva et al., 2013; Santos et al., 2014; Silva-Neto et al., 2017; Vinícius-Silva et al., 2017; Toni et al., 2020; Cooley and Vallejo-Marín, 2021; Cortés-rivas et al., 2022). Consequently, the taxonomic identification of species becomes indispensable to distinguishing the most efficient pollinators of blueberry.

Nevertheless, traditional taxonomic identification of bees and other insects normally depends on microscopic morphological characteristics or specialized molecular biology methods, which require time, high and costly technology, and the active participation of experts in the decision-making process (Jinbo et al., 2011; Gradišek et al., 2017). Moreover, the huge number of bee species and other insects is a challenge for taxonomists. It is estimated that there are about 20, 000 beespecies worldwide (Orr et al., 2020) 58% of which, about 11, 600 species of 74 genera, are able to buzz-pollinate (Cardinal et al., 2018). Due to the limitations of traditional taxonomy, the development and implementation of new technologies that also fulfill taxonomic requirements are needed (Gaston and O’Neill, 2004; Lewis and Basset, 2007).

To meet this need, the automatic classification of plants and animals based on images and sounds has been developed and tested over the last two decades (Schroder, 2002; Valliammal and Geethalakshmi, 2011; Santana et al., 2014; Yanikoglu et al., 2014), and is proving to be more practical than traditional investigations. For instance, the classification of bee species from wing images can achieve an accuracy higher than 98%, which is similar to or even higher than the classifications by human experts (Rebelo et al., 2021). Besides presenting a high accuracy, the automatic insect classification can be easily measured, tested, and replicated, relatively inexpensive and time and cost-efficient when compared to traditional manual classification (Gaston and O’Neill, 2004; Lorenz et al., 2017; Martineau et al., 2017; Rebelo et al., 2021). Nonetheless, classification based on images is difficult due to complications derived from object size and orientation, image quality, and light and/or background conditions (Gaston and O’Neill, 2004). On the other hand, sound is relatively easy to acquire and can, in principle, be picked up remotely and continuously (Gradišek et al., 2017). The automatic recognition of species based on Machine-Learning (ML), a widespread model of Artificial Intelligence, offers an automated approach for such classification tasks, and is a powerful tool for detecting and distinguishing vocal signals [e.g., (Acevedo et al., 2009; Briggs et al., 2013; Hershey et al., 2017; Stowell et al., 2019; Ribeiro et al., 2021)]. Recognizers can be used to process recordings of any acoustic wildlife species, including those of bee buzzing sounds (Gradišek et al., 2017; Nolasco et al., 2018; Terenzi et al., 2019; Cejrowski et al., 2020; Ribeiro et al., 2021). Despite the origin/purpose of buzzing sounds being completely different from that of animal vocal signals, the characteristics of buzzing sounds (frequency, amplitude, duration) are widely variable and may also differ between species and groups of bees (De Luca and Vallejo-Marin, 2013; Rosi-Denadai et al., 2018; Rebelo et al., 2021). Four studies addressed the problem of automatic bee species classification, dealing with twelve, two, four and fifteen classes, respectively (Gradišek et al., 2017; Arruda et al., 2018; Kawakita and Ichikawa, 2019; Ribeiro et al., 2021). These studies indicated that ML algorithms can generate classifiers that are able to quickly recognize bee species based solely on their buzzing sounds. However, small data sets with few bee species and/or manual audio segmentation and noise attenuation were also reported interfering with ML performance and practical applicability. Moreover, classical ML algorithms (e.g., Random Forest, Support Vector Machines, and Logistic Regression) fed by spectrogram-type data, such as the Mel-frequency cepstral coefficient (MFCC), a manually-designed summary of spectral information, represent the only method used for sound feature extraction. MFCCs can often lead to worse performance than the raw Mel spectral data from which they were derived (Stowell and Plumbley, 2014; Valletta et al., 2017). Further, the popularization of Deep Learning (DL), an emerging field of ML, has been outperforming classical ML, leading to significant advances in a wide range of bioacoustic tasks, including the recognition of animal vocalizations (Xie et al., 2019; Zor et al., 2019; Nanni et al., 2020).

Although buzzing sounds differ substantially from vocal signals both in terms of origin and functionality, automatic sound-based recognition with DL models, using multi-layered artificial neural networks, in particular convolutional neural networks (CNNs), should be especially relevant for recognizing blueberry pollinators. This may be possible because the vibrations required to efficiently extract pollen from flowers produce audible characteristic buzzing sounds that present differences among bee species (Burkart et al., 2011; Gradišek et al., 2017; Kawakita and Ichikawa, 2019; Ribeiro et al., 2021). Thus, we aimed to apply DL models to automatically identify blueberry-pollinating bees based on the characteristics of their buzzing sounds. However, neural networks, as well as traditional ML algorithms, present some limitations. Both models usually require large amounts of training data to capture the natural variability in the data to be modeled. Several data augmentation methods allow simulating overlap between multiple sound events and the resulting occlusion effects in the spectrogram. Mixup data augmentation creates new training instances by mixing pairs of features and their corresponding targets based on a given mixing ratio (Abeßer, 2020). Consequently, data augmentation can significantly enhance network performance. Thus, we also compared the performance of CNNs models combined with audio data augmentation and Mel-spectrogram with ML models at recognizing bee buzzing sounds. Due to the high efficiency and accuracy demonstrated by CNNs models in automatic sound classification in other audio domains, we expected that such models using Log Mel-Spectrogram representations and substantial data augmentation would obtain greater performance at recognizing bee species compared to classifications based on classic ML classifiers.

## 2 Materials and methods

### 2.1 Buzzing sound acquisition

The acoustic recording of bee buzzes was conducted in five highbush blueberry orchards (*V. corymbosum*) located in southern Chile (Maule and Los Ríos Regions) between the months of September and November in 2020 and 2021 . The total area of cultivated blueberry, both organic and conventional farming, per orchard ranged 3.2−141 hectares. The most common growing cultivars were Legacy, Brigitta, Duke, and Elliot. Four of the five orchards were supplemented with colonies of managed exotic bees of *Bombus terrestris* and/or *Apis mellifera* (**Table 1**).

**Table 1:** Information for the studied highbush blueberry orchards where bee buzzing sounds were acquired between the months of September and November in 2020 and 2021.

Orchard	Locality	Latitude	Longitude	Farming	Area	Cultivars	Managed bees
Agrícola Aguas Negras	Paillaco	$4^{\circ }2^{\prime }55.62^{\prime \prime }S$	$72^{\circ }45^{\prime }15.20^{\prime \prime }W$	Conventional	28 ha	Brigitta, Legacy, Elliot, Draper, Duke	*Bombus terrestris*,*Apis mellifera*
Shine Liucura	Paillaco	$40^{\circ }2^{\prime }49.89^{\prime \prime }S$	$72^{\circ }46^{\prime }49.21^{\prime \prime }W$	Organic	8.1 ha	Brigitta, Bluecrop, Coville, Elliot, Legacy	*Bombus terrestris*
Agroberries Asque	Mariquina	$39^{\circ }33^{\prime }59.4^{\prime \prime }S$	$72^{\circ }59^{\prime }28.4^{\prime \prime }W$	Organic	141 ha	Brigitta, Duke, Elliot, Legacy, Topshelf	*Apis mellifera*
Agroberries Cun Cun	Mariquina	$39^{\circ }33^{\prime }44.0^{\prime \prime }S$	$73^{\circ }02^{\prime }33.8^{\prime \prime }W$	Conventional	114 ha	Brigitta, Duke, Elliot, Legacy, Topshelf	*Apis mellifera*
Agrícola Campos Álvarez	Linares	$35^{\circ }55^{\prime }45.8^{\prime \prime }S$	$71^{\circ }29^{\prime }37.9^{\prime \prime }W$	Conventional	3.2 ha	Duke, Legacy	none

Visual searches were made for foraging bees beginning at 10:00h and ending at 18:30h as bee activity declined. To record buzzing sounds, 3−4 researchers constantly walked through the rows of blueberries hand-holding a recorder while searching for flower-visiting-bees. When a bee was observed approaching a flower, it was followed, holding a digital acoustic recorder (Zoom H4n Pro Handy Recorder) such that it was within 1−5 cm of the bee when it landed on the flower. The microphone head was pointed at the dorsal surface of the bee thorax. All bee individuals that could not be immediately identified were captured just after leaving the flower with an entomological net and placed in glass vials with ethyl acetate for taxonomic identification in the laboratory. As a consequence of that, we can assume the number of audio samples corresponds to the number of bee individuals. All sampled bee individuals were taxonomically identified at the lowest possible level by experts.

### 2.2 Acoustic pre-processing

We performed some data pre-processing before the training step in order to improve the performance of the ML models. The original sound recordings (.wav files) were manually classified and segments with bee buzzing sounds were selected. We categorized as *sonication* all the segments of buzzing sounds produced by bees vibrating blueberry flowers, and as *flight* the sounds produced by the flying displacement of the bees between flowers. Flight sounds and sonication buzzing could be easily distinguished on the recordings afterward by an experienced user since they present pronounced differences in acoustic characteristics; Ribeiro et al. (2021) showed that both *sonication* and *flight* sounds contribute equally to the training of a bee species classifier. Thus, we used both sound types together in all trials, since flight and sonication together sum a higher number of audio samples and include bee species not capable of sonicating. Recording segments with no bee sounds were not selected but were kept for subsequent steps. The set of recordings contained 518 audio samples (corresponding to 518 bees individuals) lasting on average 2 seconds, with 1,867 flight segments and 1,728 floral sonication segments (see **Table 2**). We performed these analyses using Raven Lite software (Cornell Laboratory of Ornithology, Ithaca, New York).

**Table 2:** Species richness and corresponding recording samples of flower-visiting bees of highbush blueberry cultivars in southern Chile in 2020 and 2021.

	Family	Species	N recordings	Flight segments	Sonication segments
1	Apidae	*Apis mellifera*	29	94	0
2	Apidae	*Bombus dahlbomii*	77	327	77
3	Apidae	*Bombus ruderatus*	29	150	48
4	Apidae	*Bombus terrestris*	88	387	468
5	Colletidae	*Cadeguala occidentalis*	103	371	696
6	Colletidae	*Cadeguala albopilosa*	5	12	32
7	Halictidae	*Callistochlora chloris*	8	29	13
8	Apidae	*Centris cineraria*	20	179	159
9	Colletidae	*Colletes cyanescens*	34	78	40
10	Colletidae	*Colletes nigritulus*	32	60	23
11	Halictidae	*Corynura* sp.	19	28	35
12	Colletidae	*Diphaglossa gayi*	15	50	37
13	Halictidae	*Lasioglossum* sp.	13	13	66
14	Apidae	*Manuelia postica*	12	15	5
15	Halictidae	*Ruizantheda mutabilis*	11	13	4
16	Halictidae	*Ruizantheda proxima*	23	61	25

The “ N recordings” denotes the number of audio recordings sampled per bee species; the right columns present the total number of flight and sonication segments in the audio samples.

### 2.3 Audio feature extraction

Audio feature extraction techniques transform raw audio data generated by acoustic pre-processing into features that explicitly represent properties of the data that may be relevant for ML classification. We compared two audio feature extraction techniques separately, Log Mel-Spectrogram and MFCC. The Mel Spectrogram is a way to process audio such that various DL and ML algorithms can learn from the recorded sounds. The Mel-scale is a logarithmic transformation of the signal frequency. The Mel-Spectrogram demonstrates a compressed form of sound in the time-frequency domain. This nonlinear transformation constitutes the outcome of the Short Time Fourier Transform (STFT) after the application of Mel-filters (a bank of bandpass filters with bandwidths modeled after the Mel-scale). The conversion of the frequency in hertz ( f ) to the Mel-scale is illustrated in Eq. 1.


(1) $$mel=2.595\underset{10}{\log}(1+\frac{f}{700})$$

#### 2.3.1 Data splitting

We partitioned the data set of audio samples into portions for cross-validation purposes. The data set was split into two equal-sized sets for training and testing in each replication, but unlike the work shown in (Alpaydm, 1999), the training set was separated into two pieces, with 30% used for training and 20% used for validation. Each replication splits the data as follows: 40% for training, 10% for validation and 50% for testing, for a total of 10 runs. Because each replication was created using a distinct seed, the distribution of data among them varies. We applied the Combined 5×2 Cross-validated F-Test (Alpaydm, 1999) a more reliable substitute for the 5×2cv t-test (Dietterich, 1998) for comparing the performance of supervised classification learning algorithms. The combined 5×2cv F-test reduces the drawbacks of the cross-validated t-test and has higher power and requires five replications of two-fold cross-validation.

### 2.4 Machine-learning classification

In order to relate the performance of different ML classification techniques, we evaluated our bee buzzing sounds dataset using classical ML and DL classifiers.

#### 2.4.1 Data augmentation

By definition, CNNs benefit from large training data sets, since this increases their capability of recognizing the acoustical patterns of bees. On the other hand, small training sets tend to cause overfitting bias. However, our data set is highly unbalanced, implying that some classes (bee species) present a very low number of audio samples. To overcome overfitting, we used data augmentation for the data set destined to CNN classifications. Data augmentation tends to improve the performance of ML algorithms by generating additional data for the training set of the model (Chlap et al., 2021). We then applied three data augmentation techniques to augment data during the training set of CNNs: mixup (Zhang et al., 2017) SpecAugment (Park et al., 2019a) and randomly truncated technique.

The mixup is a simple method to generate training data (Zhang et al., 2017) by mixing audio samples of two different bee species (both the feature space and the labels). If $x_{1}$ and $x_{2}$ are two different input samples (spectrograms in our case), and $y_{1}$ , $y_{2}$ their respective one-hot encoded labels, then the mixed sample and target are obtained by a simple convex combination:


$$x^{m}ix=\lambda x_{1}+\left(1-\lambda \right)x_{2}$$

$$y^{m}ix=\lambda y_{1}+\left(1-\lambda \right)y_{2}$$

where λ is a scalar sampled from a symmetric Beta distribution at each mini-batch generation:


λ≃Beta(α,α)

where α is a real-valued hyperparameter for tune.

The SpecAugment (Park et al., 2019b) is an occlusion augmentation technique, applied to Log Mel-Spectrograms. SpecAugment is applied at the mini-batch level, meaning that the same random strides are masked in all the samples of a given mini-batch. Frequency masking is applied such that f consecutive Mel frequency bins $\left[f_{0},f_{0}+f\right]$ are masked, where f is chosen from a uniform distribution from 0 to a frequency mask parameter $f^{\prime }$ , and $f_{0}$ is chosen from [0,F−f] , where F is the number of Mel frequency bins (Park et al., 2019a). SpecAugment was originally proposed in automatic speech recognition, but it has been rapidly used with success for other audio-related tasks, such as audio tagging (Park et al., 2019a).

Lastly, randomly truncated (RT) is a technique that consists of sampling *N* seconds of an audio sampling considering random parts of segments that contain buzzing sounds, instead of taking a fixed segment in each forward pass of the DL model.

#### 2.4.2 Classical machine-learning algorithms

For the classical ML approach, we chose some of the most commonly used and most successful ML classifiers at recognizing the taxonomic identity of bees by their buzzing sounds (Ribeiro et al., 2021): Logistic Regression, Support Vector Machines, Random Forest, Decision Trees and a classifier ensemble. Ensemble learning is a general meta approach to ML that seeks better predictive performance by combining the predictions of multiple models (for more details see Sagi and Rokach (2018)). Ensemble methods train multiple ML classifiers to solve the same problem and elect the class by taking a (weighted) vote of their predictions (Kuncheva, 2004).

#### 2.4.3 Deep Learning algorithms

Unlike classical ML, Deep Learning (DL), especially CNNs, allows computational models that are composed of several processing layers to learn representations of data with multiple levels of abstraction. We chose two CNNs classifiers that have reached high performance in other audio domains: EfficientNet V2 and Pre-trained Audio Neural Networks (PANNs).

EfficientNet is a family of models that are optimized for FLOPs^1^ and parameter efficiency (Tan and Le, 2019) and has shown good performance in other audio domains (Gong et al., 2021). It leverages neural architecture search to search for the baseline, named EfficientNet-B0, which is scaled up with a compound scaling strategy to obtain the family of models B1-B7. The EfficientNet V2 family is an improvement and outperforms previous models in both training speed and parameter efficiency. In this work, we used version Small of the model EfficientNet V2 family, without pre-training. The model was pre-trained with the ImageNet dataset, instead (Deng et al., 2009). ImageNet pre-trained models have been successfully used to boost the performance of CNNs models in audio classification tasks in recent years (Gwardys and Grzywczak, 2014; Müller et al., 2020; Palanisamy et al., 2020; Zhong et al., 2020; Gong et al., 2021).

PANNs is a CNN model trained on Log Mel-Spectrogram representations of AudioSet recordings (Gemmeke et al., 2017; Kong et al., 2020). AudioSet is a large-scale dataset of manually-annotated audio events that endeavors to bridge the gap in data availability between image and audio research. Using a carefully structured hierarchical ontology of 632 audio classes guided by the literature and manual curation, data from human labelers were collected to probe the presence of specific audio classes in 10 second segments of YouTube videos. PANNs architecture can be transferred to a wide range of audio pattern recognition tasks, being useful in scenarios like we have, where the total amount of data available for training is scarce.

### 2.5 Evaluation metrics

We used the following metrics to evaluate the performance of classifications generated by classifiers: Accuracy (Acc), Macro-Precision (MacPrec), Macro-Recall (MacRec) and Macro-F1 (MacF1). These metrics are determined by the classification output that comes from the confusion matrix. In this matrix, diagonal elements show the object similar to the actual label whereas off diagonals tell the misclassification information of the model.

Let i be a class from the set of classes C . Let T be test set and let c be a classifier, such that c(t)=l , where t is an element of the test set T and l∈C is a label corresponding to a class in C assigned to t by c . Let g(t) be the ground truth class label of t . In regard to the c classifier, we define:

- True Positives of class i , denoted by $TP_{i}$ , as the number of elements in T correctly labeled with class i by c , i.e., $TP_{i}=|\{t\in T|\;c\left(t\right)=g\left(t\right)=i\}|$ .
- False Positives of class i , denoted by $FP_{i}$ , as the number of elements in T that were wrongly classified by c as belonging to class i . Formally, $FP_{i}=\;|\{t\in T|\;c\left(t\right)=i\wedge g\left(t\right)\neq i\}|$ .
- False Negatives of class i , denoted by $FN_{i}$ , as the number of elements in T belonging to class i but classified by c with a label different from i , that is, $FN_{i}=|\{t\in T|\;c\left(t\right)\neq i\wedge g\left(t\right)=i\}|$ .

The above numbers are used to define traditional effectiveness measures of classifiers. These measures are: Precision, Recall and F1-score [for more detail see Ribeiro et al. (2021)].

We based performance mostly on the F1-score since classes were unbalanced and Accuracy tends to underestimate classes with a smaller number of samples in relation to those with a larger number (Steiniger et al., 2020). The $F_{1}$ measure is a combination of the precision and recall measures and is defined by Eq. 2.


(2) $$F1\left(c,i\right)=\frac{2p\left(c,i\right)r\left(c,i\right)}{p\left(c,i\right)+r\left(c,i\right)}$$

When comparing the performance of classifiers generated from distinct learning methods, it is common to use a global measure. A global measure aims at resuming the performance of the classifier over all classes in the test set. In this work we use the following global measures to compare the results of the classifiers we used: Accuracy ( Acc ) (which is equivalent to Micro-F1), Macro-Precision ( MacPrec ), Macro-Recall ( MacRec) and Macro-F1 ( MacF1 ) [for more detail see Ribeiro et al. (2021)]. The Macro measures are basically the average of the corresponding metric.


(3) $$MacF1\left(c\right)=\frac{\sum_{i=1}^{\left|C\right|}F1\left(c,i\right)}{\left|C\right|}$$

### 2.6 Baselines establishment

The majority baseline was used to compare the performance metrics of CNNs recognizers. This baseline consists in assigning all audio samples to the majority class, that is, the bee species with more audio samples: *Cadeguala occidentalis* and *Bombus terestris*. Additionally, we assigned the best ML algorithm (based on the highest Macro F1-score) as an ML baseline to compare its performance with those of CNNs. We compared the performance metrics of each CNN classifier with those from the two baselines (majority baseline and best ML classifier) using the combined 5×2 cross-validated F-test (detailed in “Data splitting” section). We assumed a significance level of α=0.05 . If the p -value was smaller than α , we rejected the null hypothesis and accepted that there is a significant difference between a pair of models.

## 3 Results

### 3.1 Characteristics of buzzing sounds

During 990 hours of sampling effort distributed among 554 non-consecutive days of 2020 and 2021 , we recorded 518 audio samples of 16 bee species visiting flowers of highbush blueberry cultivars in five orchards of southern Chile (see **Tables 1**, **2**); most, 13 species, were native Chilean bees and three were exotics. In the set of 518 audio samples, we identified 3,595 buzzing-sound segments, 1,728 were of sonication and 1,867 of flight (see **Table 2**). The distribution of samples per bee species was highly unbalanced and varied from five (*Cadeguala albopilosa* to 103 (*Cadeguala occidentalis*). The length of recordings ranged from 5 seconds to over one minute.

### 3.2 Performance of classical machine learning algorithms

**Figure 1:**
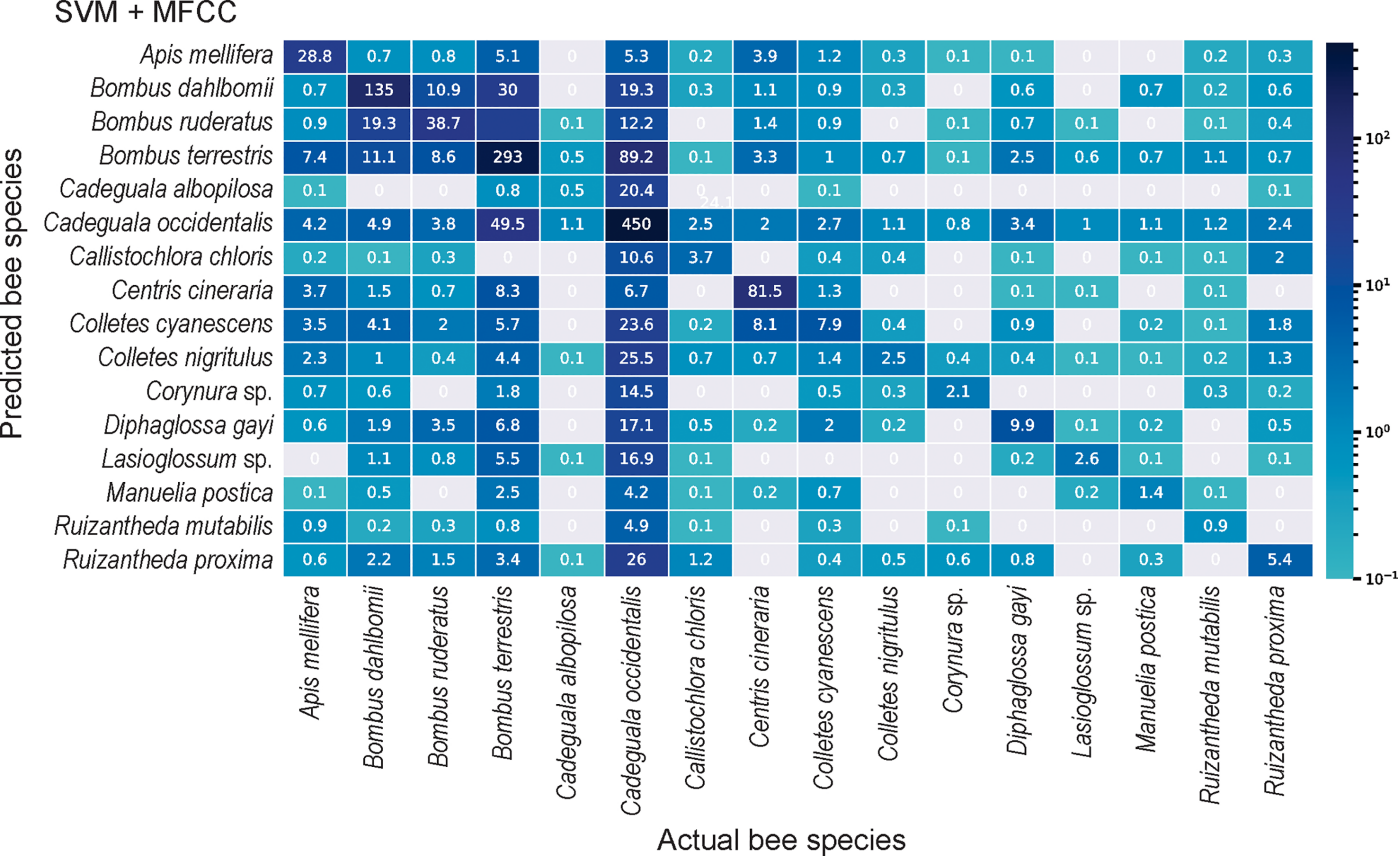
Confusion matrix showing the log-transformed number of audio segments correctly assigned to respective bee identity (diagonal elements) versus those erroneously assigned (non-diagonal elements), by the ML classifiers. SVM fed by MFCC achieved the best performance among the classical ML algorithms. Cell color represents the corresponding number (log-transformed) of audio segments predicted in a given cell, ranging from gray (zero predicted audio segments) to dark blue.

The performances (based on macro-F1 score) of the classical ML algorithms at recognizing flower-visiting bees of blueberry crops were low, ranging between 17.24 and 34.97% . However, the performances of the classical ML classifiers at recognizing bee species visiting blueberry crops depended on the algorithm employed. Support-Vector Machines (SVM) reached the highest Macro-F1 among the classical ML classifiers (**Table 3**), correctly predicting most audio samples of the majority classes (above 50% ): 61.3% of *Apis mellifera*, 67.3% of *Bombus dahlbomii*, 84.6% of *Cadeguala occidentalis* and 69.8% of *Bombus terrestris* (see **Figure 1**). However, the SVM failed to recognize most audio samples of minority classes (below 50% ): *Manuelia postica* 28.6% , *Ruizantheda mutabilis* 19.6% , and *Ruizantheda proxima* 34.2% (see **Figure 1**).

**Table 3:** Average predictive performance of different classical Machine-Learning algorithms combined with different audio feature extraction techniques (MFCC and Log Mel-Spectrogram) to recognize bee species based on buzzing sounds recorded during visits to flowers of blueberry cultivars in southern Chile.

Flight + Sonication		
Algorithms	MacF1 (%)	MacF1 (%)
	MFCC	Log Mel-Spectrogram
LR	32.52 ( ± 1.86)*^a^*	28.60 ( ± 1.07)*^a,b^*
**SVM**	**34.97 ( ± 1.52)*^a^***	**33.11 ( ± 1.65)*^a^***
RF	24.79 ( ± 0.46)*^b^*	23.66 ( ± 1.73)*^b,c^*
DTree	18.88 ( ± 2.88)*^c,d^*	17.24 ( ± 2.02)*^c,d^*
Ensemble	26.43( ± 0.73)*^c^*	21.37 ( ± 0.66)*^c^*
Mean ( ± SD)	27.51 ( ± 5.72)	24.79 ( ± 5.54)

The performances of the classical ML algorithms were measured by Macro-F1 score (MacF1) ( ± standard deviation). Bold numbers represent the best results per evaluation metric within audio feature extraction technique. Different superscript letters denote significant differences in F1-score among algorithms ( p≤0.05 , 5×2cv Combined F test).

On the other hand, the audio feature extraction technique had little effect on the performances of ML algorithms, ranging from 18.88 to 34.97% with MFCC and from 17.24 to 33.11% with Log Mel-Spectrogram. The ML algorithms presented a slightly higher performance (based on Macro-F1 score) when fed by MFCC than when fed by Log Mel-Spectrogram (**Table 3**).

### 3.3 Performance of the Deep Learning classifiers

**Figure 2:**
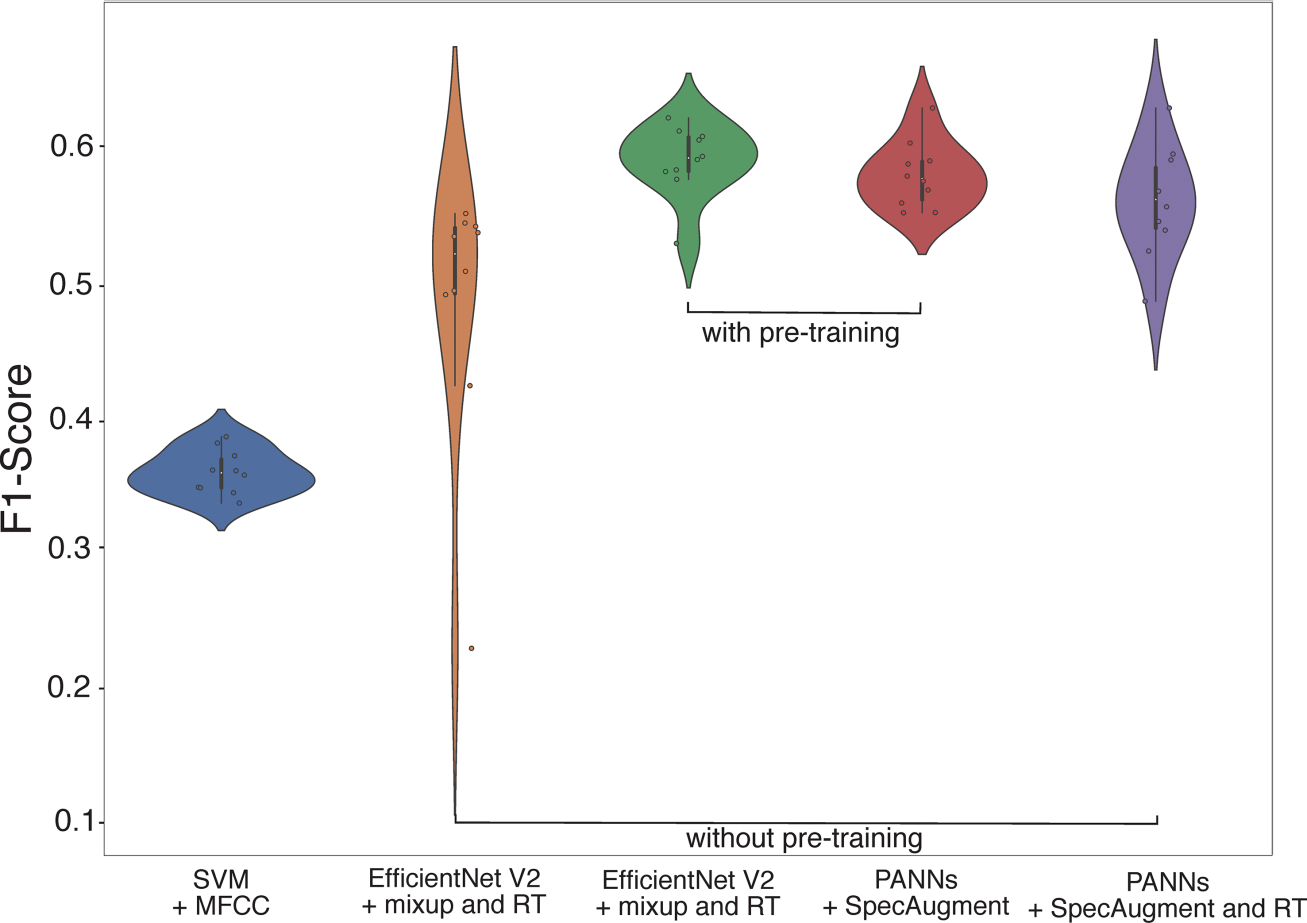
Violin plots representing the performance of the bests classical ML (SVM) and DL (EfficientNet V2 Small and PANNs) models combined with different pre-processing techniques (sound feature extraction, pre-training, and/or data augmentation) at recognizing bee species based on buzzing sounds recorded during visits to flowers of blueberry cultivars in southern Chile. Classifier performance was based on Macro-F1 score (MacF1); Each dot represents the F1 score achieved by an independent model run ( 10 runs per model, 120 epochs). Note the effect of pre-training which increased the performance of DL classifiers while reducing F1-score scattering.

Both of the tested CNNs (EfficientNet V2 Small and PANNs) reached higher performance in recognizing buzzing bee sounds than the majority baseline (assigning all the audio samples to the majority class), regardless of whether they were tested unaccompanied or combined with pre-training and/or audio data augmentation or of sampling technique (see **Table 4**). However, without data pre-processing (audio data augmentation, sampling technique, or pre-training) the CNNs did not present an evident higher performance (based on Macro-F1 score; p≤0.05 , combined 5×2cv F-test) in relation to the best classical ML classifier (SVM) (**Figure 2**). EfficientNet V2 Small overperformed SVM only when it was combined with some audio data augmentation and/or pre-training (**Figure 2**). However, PANNs without pre-training was capable of overperforming SVM, though data pre-processing also boosted its Macro-F1 score (see **Table 4**; **Figure 2**).

**Table 4:** Average predictive performance of Deep Learning models combined with an audio feature extraction technique (Log Mel-Spectrogram) to recognize bee species based on buzzing sounds recorded during visits to flowers of blueberry cultivars in southern Chile.

Methods	MacF1 (%)	MacF1 (%)
	Without Pre-training	With Pre-training
EfficientNet V2 Small	25.08 ( ± 5.25)	22.70 ( ± 6.04)
EfficientNet V2 Small + Mixup	31.91 ( ± 5.32)	43.39 ( ± 3.03) ^**^
EfficientNet V2 Small + SpecAugment	20.54 ( ± 4.61)	31.33 ( ± 2.80)
EfficientNet V2 Small + RT	37.12 ( ± 5.62)	47.39 ( ± 4.80) ^**^
EfficientNet V2 Small + Mixup + SpecAugment	14.74 ( ± 4.14)	39.32 ( ± 1.74) ^**^
**EfficientNet V2 Small + Mixup + RT**	**47.55 ( ± 9.27)**	58.04 ( ± 2.47)^**^
EfficientNet V2 Small + SpecAugment + RT	20.69 ( ± 4.88)	41.59 ( ± 4.76) ^**^
EfficientNet V2 Small + Mixup + SpecAugment + RT	16.63 ( ± 4.96)	48.48 ( ± 2.11) ^**^
**Mean ( ± SD)**	26.78 ( ± 10.55)	39.19 ( ± 7.98)
PANNs	42.66 ( ± 6.20) ^**^	35.25 ( ± 4.14)
PANNs + Mixup	52.50 ( ± 2.36) ^**^	51.95 ( ± 1.64) ^**^
PANNs + SpecAugment	52.95 ( ± 1.84) ^**^	44.85 ( ± 5.63)
PANNs + RT	46.58 ( ± 4.68) ^**^	42.55 ( ± 5.47)
**PANNs + Mixup + SpecAugment**	43.11 ( ± 3.62) ^**^	56.96 ( ± 2.30)^**^
PANNs + Mixup + RT	50.07 ( ± 1.92)^**^	52.33 ( ± 2.71)^**^
**PANNs + SpecAugment + RT**	55.00 ( ± 3.81)^**^	52.18 ( ± 3.58)^**^
PANNs + Mixup + SpecAugment + RT	35.95 ( ± 3.40)	53.33 ( ± 2.80)^**^
**Mean ( ± SD)**	47.35 ( ± 6.06)	48.67 ( ± 6.69)
Baselines		
**Methods**	**MacF1 (%)**	
Majority Class	2.98 ( ± 0.00)	
SVM	34.97 ( ± 1.52)	

The performance of the CNNs algorithms was measured by average Macro-F1 score (MacF1) (mean ± standard deviation). Bold numbers represent the best results per evaluation metric within buzz-sound. ${(}^{**})$ denotes that the performance of the algorithm is higher than the baselines (based on MacF1 score; p≤0.05 , 5×2cv Combined F test). RT, Randomly Truncated Technique; PANNs, Pretrained Audio Neural Networks.

Accordingly, pre-training increased the performance of CNNs at acoustic recognition of bee taxa (**Table 4**) by reducing the variability of F1-scores reached per model run (see **Figure 2**). The average performances of EfficientNet V2 Small and PANNs models were higher with pre-training (see **Table 4**): The Macro-F1 score of EfficientNet V2 Small ranged from 14.74%(±4.14) to 47.55%(±9.27) without pre-training and from 22.70%(±6.04) to 58.04%(±2.47) with pre-training; for PANNs they ranged from 35.95%(±3.40) to 55.00%(±3.81) without pre-training and from 35.25%(±4.14) to 56.96%(±2.30) with pre-training.

**Figure 3:**
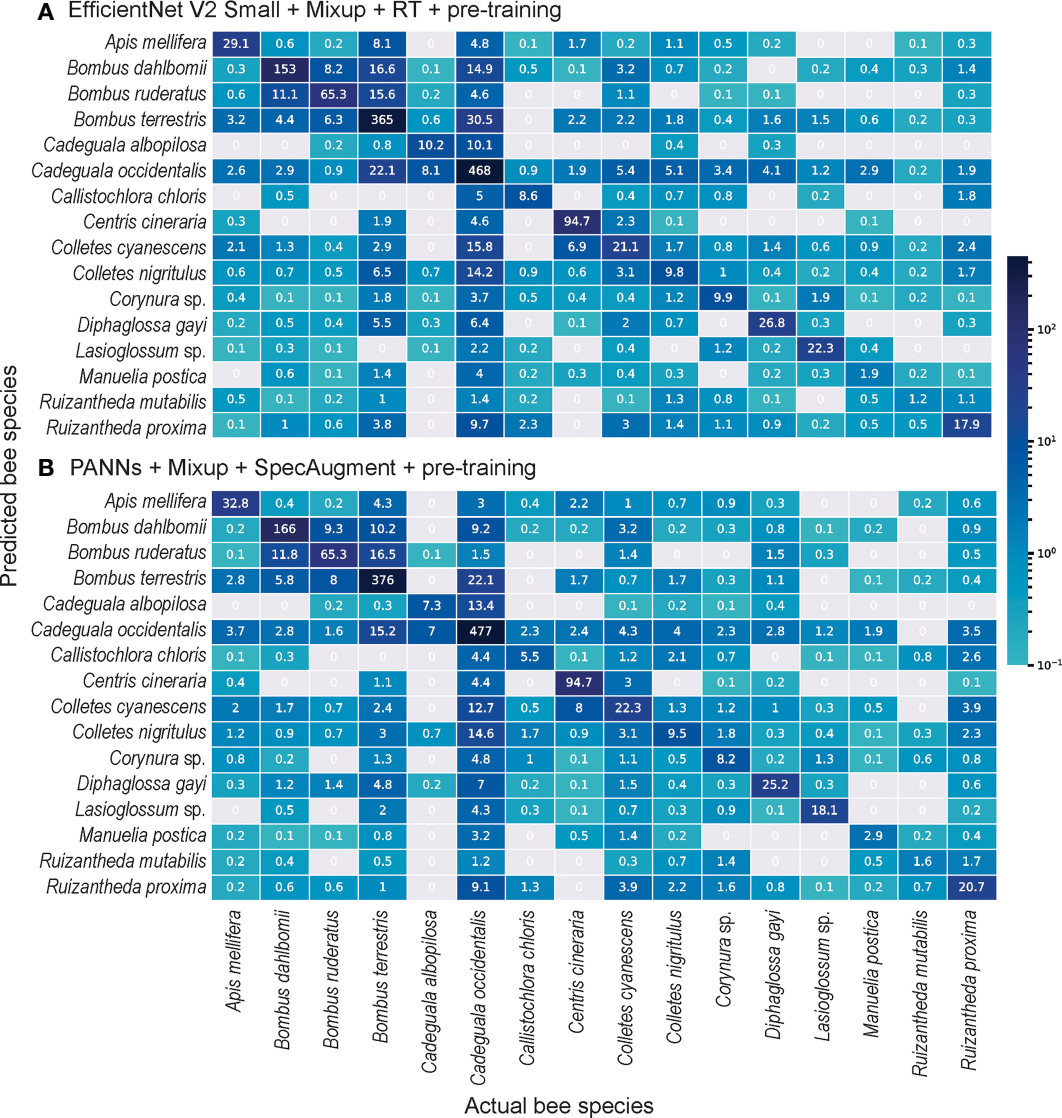
Confusion matrices showing the log-transformed number of audio samples correctly assigned to respective bee identity (diagonal elements) versus those erroneously assigned (out-of-diagonal elements) by the DL classifiers. **(A)** EfficientNet V2 Smallcombined with Mixup and RT data augmentation techniques with pre-training achieved the best performance at acoustic recognition of bees visiting flowers of blueberry crops, followed by **(B)** PANNs combined with Mixup and SpecAugment with pre-training. Cellcolor represents the corresponding number (log-transformed) of audio segments predicted in a given cell, ranging from gray (zero predicted audio segments) to dark blue.

Despite the better recognition of audio samples of the majority classes by PANNs, EfficientNet V2 Small was better at hitting the samples of minority classes. Also, EfficientNet V2 Small with Mixup RT with pre-training correctly predicted the most audio samples of the majority classes (above 50% ): 61.3% of *Apis mellifera*, 67.3% of *Bombus dahlbomii*, 84.6% of *Cadeguala occidentalis* and 69.8% of *Bombus terrestris* (see **Figure 3**). On the other hand, EfficientNetV2 Small failed to recognize most audio samples of lower represented classes (below 50% ): *Manuelia postica* 19% , *Ruizantheda mutabilis* 14.1% , *Ruizantheda proxima* 39.9% (see **Figure 3**).

Regardless of the differences above, the overall performance for acoustic recognition of bee species did not vary significantly among the CNNs architectures employed (EfficientNet V2 Small and PANNs). Despite that, EfficientNet V2 Small combined with Mixup, audio Randomly Truncated (RT), and pre-training overreached PANNs and achieved the highest Macro F1-score of 58.04%(±2.47) among all CNNs models and baselines tested (see **Table 4**).

## 4 Discussion

The studied CNNs can contribute towards automation of blueberry pollinating bee species recognition. These popular DL models reached better performances at assigning bee buzzing sounds to their respective taxa than expected by chance. However, CNNs were highly dependent on acoustic data pre-training and data augmentation to outperform classical ML classifiers at recognizing bee buzzing sounds.

### 4.1 Sound feature extraction type did not influence classical ML performance

Although the Mel-frequency cepstral coefficient (MFCC) can often lead to worse performance than the raw Mel spectral data (Stowell and Plumbley, 2014; Valletta et al., 2017), our results indicated no difference between employing MFCC and Log Mel-Spectrogram on the performance of classical ML algorithms at assigning bee buzzing sounds to the species to which they belong. MFCC is a more time-consuming method than Log Mel-Spectrogram since MFCC is a manually-designed summary of spectral information whereas Log Mel-Spectrogram involves a much simpler representation of a raw spectrogram. Despite that, MFCC has some advantages, including providing a substantially dimension-reduced summary of spectral data, which is positive for use in classical ML systems since they cannot cope with high-dimensional data (Stowell and Plumbley, 2014). However, dimension reduction necessarily implies a loss of information that could be made available for later processing and the consequent risk of discarding information that a classifier could have used. Despite MFCC being originally designed to represent human speech [24,40], which differs perceptually and from the production of bee buzzing, it can be applied to the acoustic bee recognition task with classical ML algorithms. However, our results indicated that the Mel-spectrogram, not only MFCC as previously speech (Fayek, 2016; Logan, 2000), can be a suitable sound feature extraction method for the recognition of buzzing bee species.

### 4.2 CNNs were highly dependent on pre-training and data augmentation to outperform classical ML classifiers at recognizing bees’ buzzing sounds

To our knowledge, this is the first application of CNNs to the task of acoustical classification of bee species. Despite Support-Vector Machine (SVM) being the best classical ML algorithm for bee sound recognition (Ribeiro et al., 2021), our results indicated that convolutional neural networks (CNNs) can outperform them. In fact, SVM and other classical classifiers are designed to model small variations which result in the lack of time and frequency invariance (Van Noord and Postma, 2017) which is often insufficient to cover the high-dimensional audio data of bee buzzing sounds. Therefore, CNNs become a primary choice in other applications of DL, not only for bee sound recognition recognition (Takahashi et al., 2016). In contrast to classical ML, CNNs were designed to process high-dimensional data well, which is the direct representation of raw audio data, like Log Mel-Spectrogram (Stowell and Plumbley, 2014; LeCun et al., 2015).

However, our results did not indicate that CNNs models alone overperformed classical ML, it only become evident when CNNs were combined with Log Mel-spectrogram and data augmentation techniques. In fact, CNNs can address the former limitations by learning filters that are shifted in both time and frequency (Zhang et al., 2015). However, it also generated very fast pre-training overfitting, resulting in models excessively adapted to the training set and with reduced capacity to transfer learning to validation and testing sets (Chicco, 2017). To mitigate overfitting and improve the generalization of models, we used the spectrogram augmentation technique and cross-validation to counterbalance it by generating additional pre-training audio samples and acoustic noise by applying random time-frequency masks to Log Mel spectrograms. Cross-validation is a well-known technique to deal with overfitting and was implemented in all ML classifiers here. The trained model does not overfit to a specific training subset, but rather is able to learn from each data fold, in turn (Chicco, 2017). Yet, data augmentation techniques can lead to a significant improvement in the performance of DL classifiers, but not for classical ML. Deep Learning models can take advantage of the iterative characteristic of this type of optimization, by epochs, in which the same data set can be represented in different ways for the classifiers. In practical terms, it would be like the model being exposed to different data. On the other hand, the augmented data for classical ML algorithms would be static. Therefore, we suppose that CNNs models best overperformed ML in acoustic recognition of bee species when using Mel-spectrogram information and Mixup data augmentation. Even with this improvement, however, our results indicated that the performance of CNNs is still unsatisfactory at recognizing buzzing bees in relation to ML standards (maximum F1-score 58.04%(±2.47) ). Hence, the CNNs tested here would not be the ultimate model and still have room for improvement, especially from novel Neural Networks architectures based on attention like the “transformers/perceivers” are likely to achieve higher performance for the task of bee species identification (Elliott et al., 2021; Wolters et al., 2021).

However, it is important to highlight that the two DL classifiers tested here, employ the mixup data augmentation technique slightly differently. The technique was used in the PANNs model as described in the original work, directly on the log Mel spectrogram representation. However, in the EfficientNet V2 Small model, the mixup was applied to the waveform. Based on previous experiments, we conclude that for this specific model, the application of mixup on the waveform provides better overall results.

### 4.3 Imbalanced data bias and noise corruption

In general, Machine-Learning can review large volumes of data and discover specific trends and patterns that would not be apparent to humans. To generate suitable classifications, ML models need massive resources with a considerable amount of accuracy and relevancy. However, our data set as well as other bioacoustic data sets are usually imbalanced, and with background noise (Rodrigues, 2019). Consequently, the imbalance was the main challenge to handle our data set using ML models. In-field bee audio data collection and acoustic pre-processing require domain knowledge and were exhaustive and very time-consuming: we spent 990 hours of fieldwork to record 518 audio samples, which corresponds to an average of 1.9 hours to record one sound file. Moreover, audio data collection was susceptible to bee species richness and abundance differences, thus limiting the number of samples for rare species (see also Ribeiro et al. (2021)). This not only impacts the applicability domain of the implemented ML but also influences the utility of the models for prospective use (Rodrigues, 2019). A data set is imbalanced when one class is over-represented with respect to the others, causing the model to return sub-optimal solutions due to bias in the majority class (Chicco, 2017). As a result, these classifiers tend to ignore small classes while concentrating on classifying the large ones accurately. Here, we dealt with data imbalance by employing pre-training and data augmentation (as discussed in the previous section) and measuring the performance of the classifiers based on Macro-F1 score instead of Accuracy. We employed macro-F1 since Accuracy underestimates classes with a smaller number of samples in relation to the larger ones. Macro-F1 score is considered a suited metric for an unbalanced test set because it better describes performance by class and not by sample number (Steiniger et al., 2020).

However, relating bee species performance with its respective pollination efficiency (Cortés‐Rivas et al., 2023), we found that the bees most efficient at pollinating were also the majority classes here (e.g. *B. terrestris*, *C. occidentalis*, *B. dahlbomii*). In practice, this reduces the imbalanced data bias since the majority and most hit classes are also the most efficient pollinators. Thus, we suppose that the ML algorithms are capable of recognizing well the most efficient pollinators of highbush blueberry crops in Chile.

Besides imbalanced data bias, background noise corruption was another frequent problem in our data set. However, we decided to input the original audios without noise removal or attenuation. Since noise corruption must be unavoidable in practical situations, audios without noise removal/attenuation bring more realistic model projections. In addition, by not removing noise from the input data, we also gain two functionalities: (1) we get more data for our deep neural network to train; and (2) we can train our neural network on noisy data which means that it will generalize well on noisy test data as well.

### 4.4 Consequences of automating bee recognition for blueberry fruit yields

Automating the taxonomic recognition of flower-visiting bees would be especially relevant for blueberry fruit set and size, since the quality of the pollination provided is dependent, among other factors, on the taxonomic identity of a flower visitor (Brewer and Dobson, 1969; Dogterom et al., 2000; Benjamin and Winfree, 2014; Nicholson and Ricketts, 2019). A parallel study focusing on pollinator performance and covering most of the species analyzed here revealed that only a subset of the flower-visiting bee species achieved high performance at pollinate blueberry cultivars, while others were poor pollinators or even considered flower resource thieves (Cortés-Rivas et al., 2023). Therefore, automating acoustic recognition of bee species, especially distinguishing pollinators from nectar/pollen thieves, could result in a comprehensive and powerful tool for agriculture decision-making processes. Farmers could recognize the best pollinators without needing an expert in insect taxonomy. Aware of the value of bees to crop income, farmers could be encouraged to consider the pollination perspective in their crop management, which results in the conservation of local wild bee species, thereby contributing to advances toward more sustainable and higher-yield agriculture.

In summary, we compared the performance of CNNs models at recognizing blueberry-pollinating bees with the current state-of-the-art models for bee automatic recognition. We found advantages for CNN classifiers in recognizing bee species based on their buzzing sounds over the classical ML algorithms used (Ribeiro et al., 2021). CNNs algorithms powered by a combination of transforming sound events into Mel-spectrogram images and strong data augmentation overperformed classical ML algorithms and could lead to automating the taxonomic recognition of flower-visiting bees of blueberry crops. As far as we know, the use of DL classifiers for bee taxa identification based on respective buzzing sounds has not been reported previously. However, there is still room to improve the performance of DL models. Further studies, focusing on recording samples for poorly represented classes, and/or applying algorithms that can perform more complex processing tasks like unsupervised learning systems, could help to achieve better classification results.

## Data availability statement

The raw data supporting the conclusions of this article will be made available by the authors, without undue reservation.

## Author contributions

NS, FM, TR, and JM-N contributed to conception and design of the study. VM and JM-N carried out the experiment. AF organized the database. AF performed the data and statistical analysis. FM and JM-N wrote the first draft of the manuscript. TR, AF, and NS wrote sections of the manuscript. All authors contributed to the article and approved the submitted version.

## Footnotes

1 Acronym for Floating Operations per Second.
